# The Impact of Different Types of Shift Work on Blood Pressure and Hypertension: A Systematic Review and Meta-Analysis

**DOI:** 10.3390/ijerph18136738

**Published:** 2021-06-23

**Authors:** Sara Gamboa Madeira, Carina Fernandes, Teresa Paiva, Carlos Santos Moreira, Daniel Caldeira

**Affiliations:** 1 Instituto de Saúde Ambiental (ISAMB), Faculdade de Medicina, Universidade de Lisboa, 1649-026 Lisbon, Portugal; 2 Family Health Unit Mactamã, Administração Regional de Saúde de Lisboa e Vale do Tejo, 2745-862 Lisbon, Portugal; 3 Escola Nacional de Saúde Pública, Universidade Nova de Lisboa, 1600-560 Lisbon, Portugal; fernandes.carina@gmail.com; 4 Neurology Department, Hospital das Forças Armadas, 1649-020 Lisbon, Portugal; 5 Sleep Medicine Center (CENC), 1070-068 Lisbon, Portugal; teresapaiva0@gmail.com; 6 Comprehensive Health Research Center (CHRC), Nova Medical School, Universidade Nova de Lisboa, 1169-056 Lisbon, Portugal; 7 Medicine Clinic I, Faculdade de Medicina, Universidade de Lisboa, 1649-028 Lisbon, Portugal; carlos.moreira@medicina.ulisboa.pt; 8 Cardiology Department, Hospital de Santa Maria/Santa Maria University Hospital—Centro Hospitalar Universitário Lisboa Norte (CHULN), 1649-028 Lisbon, Portugal; dgcaldeira@hotmail.com; 9 Laboratory of Clinical Pharmacology and Therapeutics, Faculdade de Medicina, Universidade de Lisboa, 1649-028 Lisbon, Portugal; 10 Centro Cardiovascular da Universidade de Lisboa (CCUL), CAML, Faculdade de Medicina, Universidade de Lisboa, 1649-028 Lisbon, Portugal

**Keywords:** cardiovascular disease, blood pressure, occupational health, work schedule, permanent shift, rotating shift, night shift, systematic review

## Abstract

Shift work (SW) encompasses 20% of the European workforce. Moreover, high blood pressure (BP) remains a leading cause of death globally. This review aimed to synthesize the magnitude of the potential impact of SW on systolic blood pressure (SBP), diastolic blood pressure (DBP) and hypertension (HTN). MEDLINE, EMBASE and CENTRAL databases were searched for epidemiological studies evaluating BP and/or HTN diagnosis among shift workers, compared with day workers. Random-effects meta-analyses were performed and the results were expressed as pooled mean differences or odds ratios and 95% confidence intervals (95% CI). The Newcastle–Ottawa Scale was used to assess the risk of bias. Forty-five studies were included, involving 117,252 workers. We found a significant increase in both SBD and DBP among permanent night workers (2.52 mmHg, 95% CI 0.75–4.29 and 1.76 mmHg, 95% CI 0.41–3.12, respectively). For rotational shift workers, both with and without night work, we found a significant increase but only for SBP (0.65 mmHg, 95% CI 0.07–1.22 and 1.28 mmHg, 95% CI 0.18–2.39, respectively). No differences were found for HTN. Our findings suggest that SW is associated with an increase of BP, mainly for permanent night workers and for SBP. This is of special interest given the large number of susceptible workers exposed over time.

## 1. Introduction

Hypertension (HTN) is a major preventable cause of cardiovascular diseases (CVDs) and all-cause mortality in the European continent, with an overall prevalence of 30–45% [1]. There is a relationship between blood pressure (BP) and CVD events [2], and BP decrease in hypertensive patients has shown to improve the prognosis [3]. Guidelines on CVD prevention stress the importance of a holistic approach, including non-traditional risk factors such as socioeconomic status and occupational factors [4]. Shift work (SW) plays an important role in the “24/7” modern societies, involving about 20% of the European and the American workforces [5]. However, this work arrangement frequently disrupts sleep-wake cycle and circadian rhythms, which may affect cardiovascular function including BP. Since shift work is a growing societal trend and high BP a leading risk factor for cardiovascular diseases, it is crucial to clarify the potential impact of shift work, especially when robust data is lacking. The single previous systematic review in this topic focused only on the HTN risk and used heterogeneous definitions for HTN diagnosis and simplistic SW categorization [6]. Therefore, we aimed to determine not only the HTN risk but also the magnitude of BP change among shift workers in comparison with day workers.

## 2. Materials and Methods

This systematic review was conducted in accordance with PRISMA (Preferred Reporting Items for Systematic Reviews and Meta-Analysis) guidelines [7] and its protocol was registered (Available online: https://osf.io/m47qc (Accessed on 24 May 2021)).

### 2.1. Literature Search and Selection

A literature search was performed by personnel experienced in designing strategies for systematic reviews in health sciences databases. The search was performed in MEDLINE, EMBASE and The Cochrane Library electronic database (CENTRAL), on 18 February 2019. There were no limits regarding year of publication, language, study design or geographic origin. Animal studies were excluded. The search strategy is detailed on the supplementary material (Table S1). Two reviewers (SGM and CF) independently evaluated the title and abstract of the retrieved papers to determine if these met the inclusion criteria, using a pre-piloted form. Studies fulfilling the inclusion criteria and those uncertain were analyzed in full-text independently by the two reviewers. At this stage we only considered articles published in English and the reasons for exclusion were recorded. Abstracts and conference papers were excluded. Disagreements were solved through consensus or using a third party (DC).

### 2.2. Inclusion Criteria

We included studies that reported data about BP values and/or diagnosis of HTN in both shift workers and a control group of day workers. We were lenient and broad regarding the definition of shift work, therefore we considered any shift provided if represented a nonstandard schedule, excluding long work hours (e.g., weekend work). If studies reported BP values, we sought the systolic and/or diastolic BP mean values and standard deviation (or other measurement of variability), in both groups. Data from linear regression models on BP values (mmHg), reporting a β coefficient and 95% CI, were also considered. HTN diagnosis was recorded when it was established using the cut-off values of the current European Guidelines (i.e., systolic BP ≥ 140 mmHg and/or diastolic BP ≥ 90 mmHg in office) [1]. HTN diagnosis was also considered when the subject was under anti-hypertensive medication. Studies in which this diagnosis relied on subjects’ self-report or those having other HTN definition thresholds were excluded. Data from binary logistic regression models, reporting estimation of risk (e.g., odds ratio) were included. Studies enrolling exclusively special populations (e.g., pregnant women or clinical populations) and laboratory protocols were excluded since our focus was on “real-life” settings. Additionally, when different papers included, either totally or partially, the same subjects, we selected the study which more accurately and comprehensively answered our research question. For more details see the supplementary material (Table S2).

### 2.3. Data Extraction

Data was independently extracted from the included studies by two reviewers (SGM and CF) into a standardized form. Disagreements were solved through consensus. The following data were extracted: study design and follow-up (for longitudinal studies), occupational setting, sample size, mean age, sex, shift work schedule definition and source of information and method of BP assessment. For outcomes, systolic and diastolic BP mean and standard deviation or standard error, HTN diagnosis, effect size measurements with 95% confidence intervals and confounding variables. Adjusted risk estimates were preferred. When more than one regression model was presented, the one that best fitted our research question was included.

### 2.4. Methodologic Quality Assessment

The methodologic quality assessment was also performed independently by two reviewers (SGM and CF). Included studies were graded according to the adequate version of the Newcastle–Ottawa Quality Assessment Scale (NOS) [8,9]. This tool evaluates three dimensions (selection, comparability and outcome), distributed across eight items. A maximum of one point for each item within the “Selection” and “Outcome” categories and maximum of two points for “Comparability” can be given. Higher scores represent a higher methodologic quality; less than 5 points was considered as low quality/high risk of bias [9]. For outcome assessment in cohort studies, the adequate follow-up was defined as 5 years, based on the dose-response relationship between shift work and cardiovascular outcomes suggested in previous studies [10].

### 2.5. Data Analysis

For analysis purpose, we defined categories of SW considering 4 types: permanent night shifts (PN), rotational shifts including nights (R + N), rotational shifts without nights (RN) and an additional category for the remainder (NS; “Not Specified”). Studies that included several types of SW (e.g., permanent night workers and rotational shifts including nights) were considered independent entries and included in independent meta-analyses.

Pooled mean difference and 95% CI were estimated for continuous outcomes (systolic BP and diastolic BP) to quantify the difference in means between each SW type and controls. Pooled odds ratio (OR) and 95% CI were determined for the dichotomous variable (HTN diagnosis), through random-effects models. The statistical analyses were performed using RevMan 5.4 software (The Nordic Cochrane Centre, The Cochrane Collaboration). Heterogeneity of the pooled effect size estimates was assessed through the I^2^ statistic to quantify the proportion of the total variation across studies that resulted from heterogeneity rather than chance. Publication bias was assessed through visual inspection of funnel plot asymmetry (see supplementary material-Figure S1) and, also, by Egger test.

Whenever more than ten studies were involved in the meta-analysis of continuous outcomes variables (i.e., SBP and DBP) [11] a meta-regression analysis was performed in order to assess if specific factors (covariates) influence the magnitude of the estimate of effect estimate across studies [11,12]. Similarly to what has been conducted in previous studies on this topic [13], we include covariates related to participants characteristics such as sex (proportion of males) and age (mean values) but also important cardiovascular risk factors such as smoking (proportion of smokers) and body mass index (BMI; average values). We performed univariate and multivariate meta-regression analysis.

## 3. Results

### 3.1. Search Results

**Figure 1:**
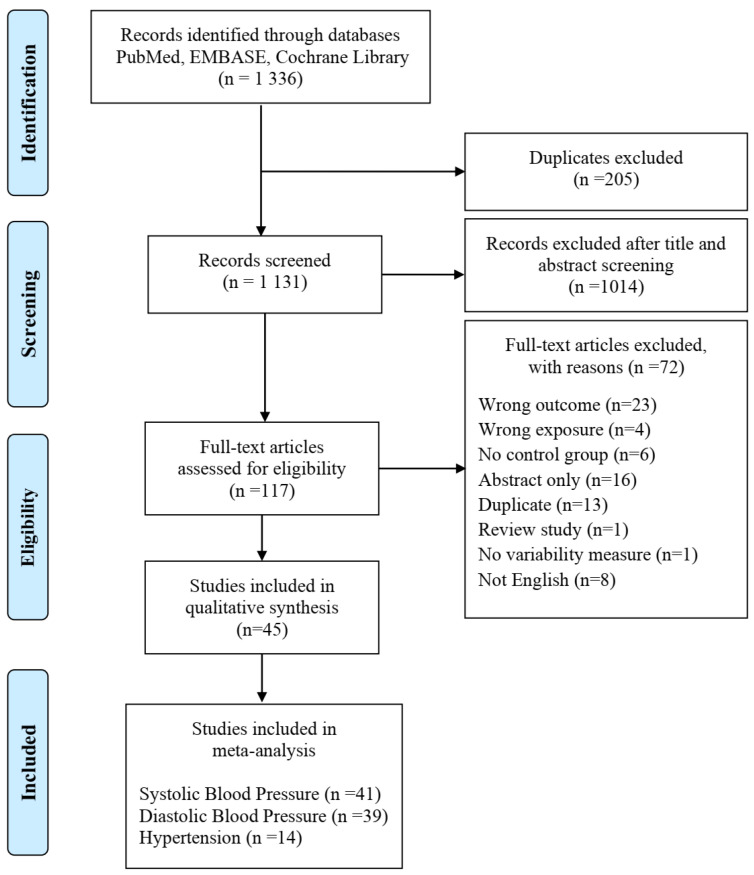
PRISMA flow diagram of literature search, screening and eligibility of the included studies in the meta-analysis.

Of the 1336 articles retrieved from the electronic database search, 117 underwent full-text assessment. At full-text appraisal, 72 studies were excluded (Figure 1). At this stage, retrieval of conference abstracts, lacking a full-text article, lead to their exclusion (labelled as “abstract only”). When the same population was used in different studies, only one of the studies was selected (the exclusion was labelled as “duplicate”; more detailed information is provided in the supplementary material-Table S2). Forty-five independent studies met the inclusion criteria. Of these, 41 were included in the meta-analysis for systolic BP, 39 for diastolic BP and 14 for HTN diagnosis (Figure 1). A total of 117,252 workers were implicated, 46,345 of which shift workers (SWs) and 70,907 daytime workers (DWs).

### 3.2. Study Characteristics

Main characteristics of the 45 included studies [14,15,16,17,18,19,20,21,22,23,24,25,26,27,28,29,30,31,32,33,34,35,36,37,38,39,40,41,42,43,44,45,46,47,48,49,50,51,52,53,54,55,56,57,58] are presented in Table 1.

Most studies had a cross-sectional design or provided only cross-sectional information. Three studies provided longitudinal data, two being retrospective cohorts [17,36] and one a prospective cohort [49]. The follow-up periods ranged from 10 to 31 years. Most studies were settled in Asia (*n* = 21), mostly in Japan, followed by Europe (*n* = 13), America (*n* = 9) and, lastly, Africa (*n* = 2). Industry was the most frequent occupational setting (*n* = 25), followed by transportation (*n* = 4) and nursing staff (*n* = 4). Nevertheless, the specific job performed by the participants was not always explicit, both for SWs and DWs. In six studies, the authors highlighted that the SWs were mainly blue-collar workers (e.g., machine operators) while DWs were mainly white-collar (e.g., administrative). Sample sizes ranged from 47 to 26,463 participants. Most studies included only male workers (*n*= 26), while 9 studies addressed only females and 10 studies incorporated both sexes. Overall, the participants’ mean age was 39.61 years, specifically, 39.64 for SWs and 39.58 for DWs.

For exposure assessment, most studies used questionnaires or interviews (*n* = 35) and the remainder used company records (*n* = 10). The definition of SW was very heterogeneous. Given the original description of SW schedules, we categorized the shift workers according to the influence of work schedule in the night-time and, as a result, the potential impact on sleep and circadian system. Three categories emerged: permanent night shifts (PN; *n* = 14), rotational shifts including nights (R + N; *n* = 28) and rotational shifts without nights (R-N; *n* = 4). In some cases, the type of schedule was not well explicit [14,22,28,37] or the population of SWs resulted from a combination of different schedules [15,48,50,56]. Such cases were labelled as a fourth category “Not Specified” (NS; *n* = 8). Of note, studies that included different types of SW (e.g., permanent night workers and rotational shifts including nights) compared to the same population of DWs were considered independent entries and included in independent meta-analyses. This provided segregate results according to the type of SW, with a more homogeneous exposure within groups.

Most studies provided more than one outcome of interest. A frequent combination was systolic BP and diastolic BP (*n* = 31), but also systolic BP, diastolic BP and HTN diagnosis (*n* = 8), with 4 studies accounting just for HTN and only 2 studies reporting systolic BP and HTN. Three studies provided data from ambulatory blood pressure monitoring [56,57,58]. Information regarding drug treatment with antihypertensive drugs was not reported or taken into consideration in most studies. A minority of studies had controlled the outcomes of interest for confounding factors (*n* = 13). Age was a ubiquitous adjusted variable. Other variables included lifestyle factors (e.g., smoking, alcohol and exercise) and occupational characteristics (e.g., job duration). Only one study [25] adjusted for sleep disturbances, whereas none controlled for sleep duration or deprivation, sleep quality or individual chronotype.

### 3.3. Risk of Bias

The Newcastle–Ottawa Scale (NOS) was used to evaluate the risk of bias of the included studies. The average NOS score was 5.6 points (median = 5; interquartile range = 2.25) with eleven studies scoring below 5 (low quality/high risk of bias) [9]. These eleven studies contributed to SBP and DBP results and only one for HTN. All included studies scored in the outcome and exposure ascertainment items since we excluded self-reported outcomes and exposure data derived from questionnaires or records. Therefore, the weakest dimension was comparability, with a minority of studies controlling the results of interest for confounding factors. All the included cohorts had an adequate follow-up period. The total NOS score for each included study is presented in Table 1. More details about the risk of bias of individual studies are shown in supplementary material (Table S3).

### 3.4. Effect of Shift Work on Systolic Blood Pressure (SBP)

**Figure 2:**
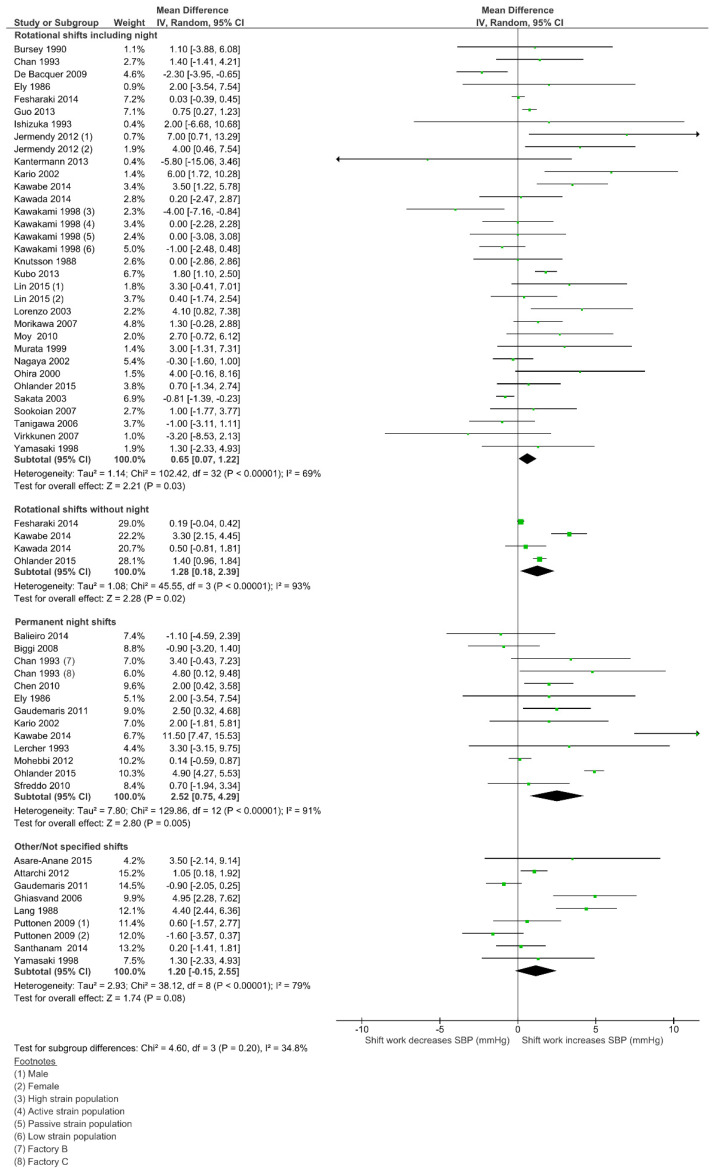
Forest plot showing the potential impact of the different shift work types in systolic blood pressure (SBP).

Weighted mean differences and 95% CI for systolic BP (SBP), according to the SW type, are shown in Figure 2. Permanent night work had the highest estimate, with a 2.52 mmHg increase on SBP (95% CI 0.75–4.29; I^2^ = 91%; 12 studies; 29,923 participants). A positive effect was also found among rotational shifts without night work, with a 1.28 mmHg increase (95% CI 0.18–2.39; I^2^ = 93%; 4 studies; 31,805 participants). Within the most common exposure, rotational shifts including night work (28 studies; 81,687 participants), the increase on SBP was 0.65 mmHg (95% CI 0.07–1.22; I^2^ = 69%). The “Not Specified” group had an estimate that did not reach statistical significance (1.20 mmHg; 95% CI 0.15–2.55; I^2^ = 79%; 8 studies; 10,548 participants). Subgroup differences were not statistically significant (*p* = 0.20) and there was no evidence of publication bias according to the Egger test (*p* = 0.418). Meta-regression analysis did not find a significant modifier effect on the mean difference of SBP for any of the covariates analyzed (sex, age, smoking and BMI) (see supplementary material for full results—Tables S4 and S5).

### 3.5. Effect of Shift Work on Diastolic Blood Pressure (DBP)

**Figure 3:**
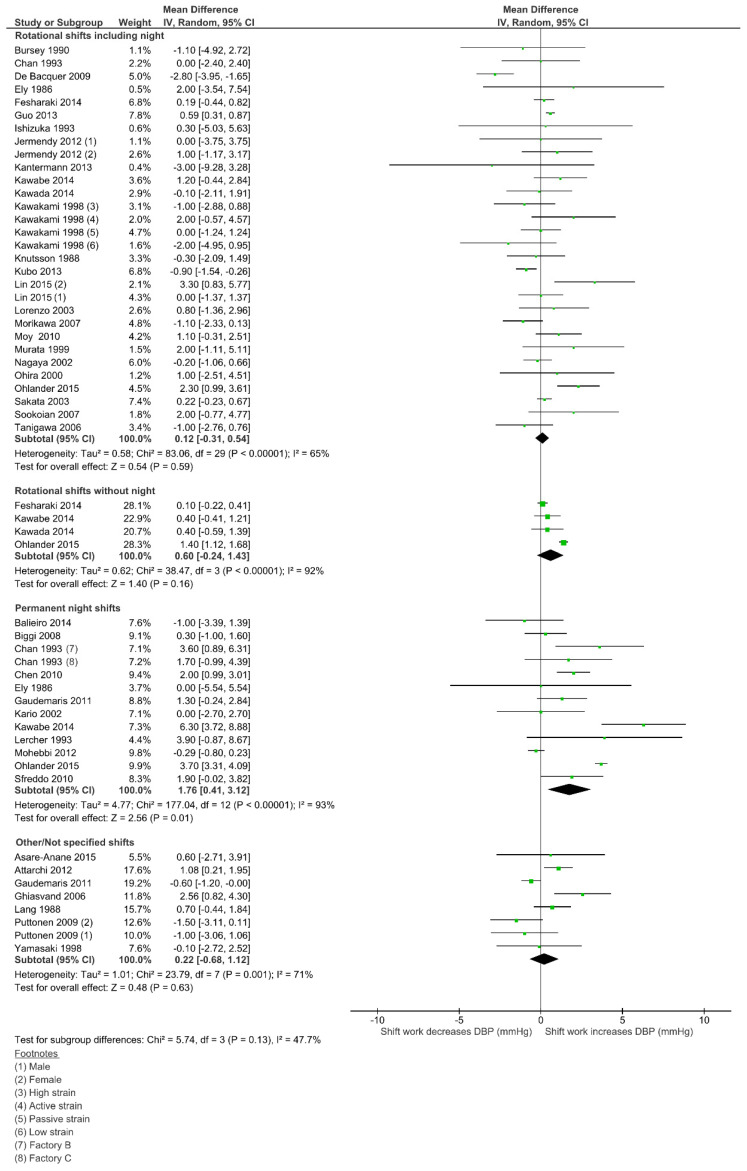
Forest plot showing the potential impact of the different shift work types in diastolic blood pressure (DBP).

Weighted mean differences and 95% CI for diastolic BP (DBP), according to the SW type, are shown in Figure 3. As for SBP, the permanent night work had the highest estimate, with a 1.76 mmHg increase on DBP (95% CI 0.41–3.12, I^2^ = 93%; 12 studies; 29,923 participants). In fact, this was the only subgroup that reached statistical significance on DBP. As well as for SBP, the rotational shifts without nights was the second highest (0.60 mmHg; 95% CI 0.24–1.43; I^2^ = 92%; 4 studies; 31,805 participants), followed by rotational shifts including night work (0.12 mmHg; 95% CI 0.31–0.54; I^2^ = 65%; 25 studies; 81,195 participants) and, finally, the “Not Specified” group (0.22 mmHg; 95%CI 0.68–1.12; I^2^ = 71%; 7 studies; 7385 participants). No subgroup differences were statistically significant (*p* = 0.13) and there was no evidence of publication bias (Egger test *p* = 0.447). Meta-regression analysis did not find a significant modifier effect on the mean difference of DBP for any of the covariates analyzed (sex, age, smoking and BMI) (see supplementary material for full results—Tables S4 and S5).

### 3.6. Effect of Shift Work on Hypertension (HTN)

**Figure 4:**
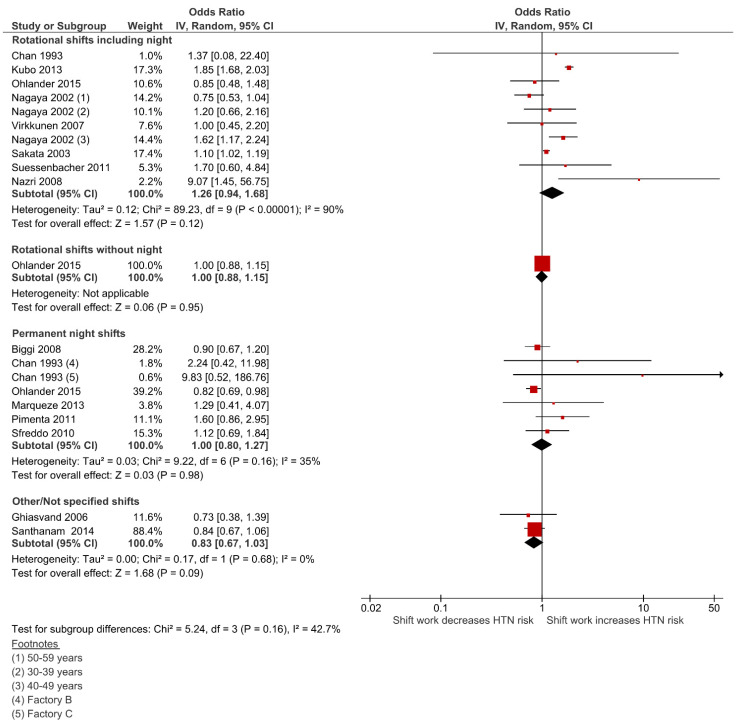
Forest plot showing the potential impact of the different shift work types in hypertension (HTN).

The pooled analysis showed that none of SW types were significantly associated with neither an increase nor a reduction in the risk for HTN diagnosis (Figure 4). The rotational shifts including night work, the most frequent SW type (8 studies; 33,716 participants), had the highest estimate with an increased risk of HTN by 26%, however this was not statistically significant (OR = 1.26; 95% CI 0.94–1.69; I^2^ = 90%). Permanent night work revealed a neutral effect on HTN risk (OR = 1.00; 95% CI 0.80–1.27; I^2^ = 35%; 6 studies; 17,075 participants), as well as rotational shifts without nights (OR = 1.00; 95% CI 0.88–1.15; 1 study; 21,577participants) and the “Not Specified” group (OR = 0.83; 95% CI 0.67–1.03; I^2^ = 0%; 2 studies; 3586 participants). No subgroup differences were statistically significant (*p* = 0.16) and there was no evidence of publication bias (Egger test *p* = 0.957).

## 4. Discussion

### 4.1. Main Findings

The main findings of this review, based on 45 independent studies which evaluated 46,345 shift workers against 70,907 day workers, were: (1) night workers had a statistically significant increase in both systolic and diastolic BP values; (2) rotational shift workers, both with and without night work, had a significant increase only in systolic BP; (3) the magnitude of the effect was small, ranging from 0.65 to 2.52 mmHg, and the larger upper bound of the pooled confidence intervals was 4.29 mmHg. This might seem as not clinically significant, however, it should be considered in susceptible populations continuously exposed over a considerable period of time, as a possible contributing factor for the development of HTN and/or for the need of more intensive drug treatment. Moreover, it was clearly demonstrated that the SW effect on BP values, although modest, is more consistent for SBP. This may be of special relevance considering that SBP has a major impact on CVD events [2]. Concerning HTN risk, we did not find a significant increase in any of the SW types assessed. This finding differs from the single previous meta-analysis in this topic [6], which found a greater risk among shift workers in cohort studies (OR = 1.31; 95% CI 1.07–1.60) and an almost statistically significant increase among cross-sectional ones (OR = 1.10; 95% CI 1.00–1.20). Differences in these results can be explained by broader inclusion criteria in the previous review such as wider HTN definitions (e.g., metabolic syndrome thresholds of 130/85 mmHg), specific populations (e.g., sleep-disorder breathing patients and pregnant women) and different classifications of shift work types. Also, age is a major determinant for HTN [1] and the low average age of the included participants in our review (i.e., below 40 years) may have conditioned a low incidence of HTN, where differences between groups were not apparent. Since study subjects included in this systematic review were relatively young, the risk of hypertension in elderly shift workers may be increased. Further research will be needed concerning this aspect.

### 4.2. Overall Limitations of Included Studies

This is a systematic review with meta-analysis of study-level data, thus, our results are limited by the potential bias and intrinsic methodological limitations of the studies included. In fact, a major limitation of our review is related with the scarcity of adequate longitudinal data. This precludes not only the control for selection bias (the so-called healthy shift worker effect) but also the determination of a time sequence and a dose-response relationship which, in turn, hinders the assumption of causality. The “healthy shift worker effect” refers to the tendency for individuals with poorer health more likely quit shift work (survivor effect) or avoid it in the first place (hire effect) [59], resulting in an underestimation of the effects of shift work. On the other hand, the frequent higher payment for the same job, when performed outside the standard hours, can lead to a selection of lower socioeconomical status workers for SW. This is an important consideration given that lower socioeconomical conditions are associated to higher CVD risk [4] and few studies controlled for these variables. Furthermore, jobs which require SW frequently entail the performance of tasks with a higher physical strain. This alone may be associated to a higher risk for HTN, and few studies controlled for this specific issue. Indeed, one of the few which did, found a higher influence of physical strain than that of SW in the SBP and HTN [55]. Considering that the main mechanisms involved in the health consequences of shift work are unhealthy behaviors, sleep disturbance and circadian misalignment [59], only the first was assessed and controlled for in adjusted analyses. Sleep deprivation is commonly associated with SW and, in itself, represents a recognized cause for increased HTN risk [60] but almost no study evaluated and controlled for sleep duration and quality parameters. The same applies to the chronotype assessment, as a measurement of circadian entrainment, which can play a role in SW adaptation [61]. As diurnal creatures, human circadian system enables us to anticipate the light/dark cycle, ensuring optimal physiological functioning during the active day and restorative functioning during sleep. A healthy circadian rhythm of BP includes a considerable decrease during sleep, known as “dipping”, that can be altered with shift work [62]. This confers biological plausibility for our results that revealed a higher risk of increased blood pressure among permanent night workers. It also highlights the importance of assessing BP through ambulatory blood pressure monitoring given the high CVD prognostic value of sleep-time BP [1].

### 4.3. Strengths and Limitations

To the best of our knowledge, this is the first systematic review with meta-analysis that assessed the impact of different types of SW on BP values, both systolic and diastolic. This is of special interest since CVD events have a continuous and proportional relationship with BP values [2]. Furthermore, this approach allowed the inclusion of studies which main outcome was not hypertension, but nevertheless provided BP measurements. As for HTN risk assessment, we assumed a strict and conservative approach by only considering the current HTN thresholds and excluding self-reported outcomes. Another innovative aspect was the division in specific types of SW, according to night-time work. This aimed to counteract the notoriously heterogenous nature of the SW definition and operationalization, allowing for more homogenous exposed groups concerning the circadian system and more precise results. Moreover, this strategy allowed for the same study providing data for more than one meta-analysis.

On the other hand, when we segregated the results into SW types, some groups resulted in too few studies. High levels of heterogeneity among pooled results were found. This may be due to the wide heterogeneity in the work settings and tasks performed in the included studies. In fact, although we have tried to mitigate the SW variability, even our SW types may encompass different working times, schemes, speed and direction of rotation. Additionally, the duration and intensity of the SW exposure (e.g., average number of shifts) may be implicated, since most studies did not provide any information about these features. Another possible limitation is a geographic bias, with almost half of the studies developed in Asia.

## 5. Conclusions

There is sufficient evidence for a potential link between permanent night shift work and an increase in blood pressure values. Regarding rotational shift work, both including nights or not, the evidence is only for an increment in systolic BP. As for hypertension, no increased risk was found. Although the effect on BP values was rather small, this can be of special interest in borderline situations or in susceptible populations with concurrent cardiovascular risk factors. Occupational health services may play an important role in limiting shift work health consequences by promoting healthy behaviors, while closely monitoring the more vulnerable workers. Considerations about circadian human physiology could support the design of least detrimental work schedules and select more adequate workers for certain shifts, according to their own individual chronotype. To accurately define the impact of shift work on blood pressure, interventional and longitudinal studies with appropriate follow-up are needed, which should include comprehensive shift work descriptions, continuous BP monitoring and, also, adjustment for relevant lifestyle, occupational and sleep parameters.

## Figures and Tables

**Table 1:** Main characteristics of the 45 included studies.

Author Year	Design	Country	Population	Sex	Shift Work	Sample Size (SWs/DWs)	Mean Age ^i^ (SWs/DWs)	Outcome	Outcome Adjustments	NOS
Asare-Anane 2015 [14]	CS	Ghana	cocoa industry	F&M	NS	113/87	42.0/40.3	SBP DBP	No	4
Attarchi 2012 [15]	CS	Iran	tire manufacturing factory	M	NS	88/76	38.5/40.2	* SBP * DBP	* age, BMI, smoking, salt, exercise, family HTN, job duration	8
Balieiro 2014 [16]	CS	Brazil	bus drivers	M	PN	81/69	44.0/46.7	SBP DBP	No	4
Biggi 2008 [17]	CH (76-07)	Italy	street cleaning and waste collection	M	PN	331/157	47.0/42.3	* HTN ** SBP ** DBP	* age, job company and branch, study period ** plus smoking and alcohol	8
Bursey 1990 [18]	CS	UK	nuclear fuel factory	M	R + N	57/57	50/50	SBP DBP	No	5
Chan 1993 [19]	CS	Singapore	electronics industry	F	R + N PN	R + N 55/75 PN ^B^ 73/63 PN ^C^ 58/59	R + N 28/30 PN ^B,C^ NotR	SBP DBP HTN	No	4
Chen 2010 [20]	CS	Taiwan	semiconductor manufacturing	F	PN	561/656	32.7/34.9	SBP DBP	No	4
De Bacquer 2009 [21]	CS	Belgium	nine companies and public administration	M	R + N	309/1220	44.7/43.1	SBP DBP	No	6
Gaudemaris 2011 [22]	CS	France	nursing staff	F	PN NS	PN 149 NS 1802/1863	NotR	SBP DBP	No	6
Di Lorenzo 2003 [23]	CS	Italy	chemical industry	M	R + N	185/134	48.7/48.9	SBP DBP	No	6
Ely 1986 [24]	CS	US	police officers	M	R + N PN	R + N 41 PN 80/156	R + N 37.4 PN 38.1/40.0	SBP DBP	No	6
Ohlander 2015 [25]	CS	Germany	car manufacturing	F&M	R + N R-N PN	R + N 198 R-N 9572 PN 3568/12,005	R + N 40.0 R-N 38.3 PN 41.4/37.8	SBP DBP * HTN	* age, sex, BMI, lipids, smoking, alcohol, exercise, sleep disorders, job status, noise, heat, social disruption	8
Fesharaki 2014 [26]	CS	Iran	steel and polyacryl companies	M	R + N R-N	R + N 4050 R-N 597/3966	R + N 41.62 R-N 43.31/41.33	* SBP * DBP	* age, BMI, education, work experience, marital status	8
Guo 2013 [27]	CS	China	motor corporation	F&M	R + N	9118/17,345	62.4/64.22	SBP DBP	No	6
Ghiasvand 2006 [28]	CS	Iran	railroad company	M	NS	158/266	46.4/38.69	SBP DBP *HTN	* age, BMI, eating habits	6
Ishizuka 1993 [29]	CS	Japan	machine plant	M	R + N	38/21	31.6/36.9	SBP DBP	No	5
Jermendy 2012 [30]	CS	Hungary	multiple occupations	F&M	R + N	M 54/67 F 180/180	M 42.2/42.5 F 44.5/42.9	SBP DBP	No	4
Kantermann 2013 [31]	CS	Belgium	steel factory	M	R + N	32/15	39.5/45.0	SBP DBP	No	4
Kawabe 2014 [32]	CS	Japan	12 large companies	F&M	R + N R-N PN	R +N 243 R-N 1017 PN 73/3094	R + N 40.1 R-N 37.9 PN 50.8/42.6	SBP DBP	No	5
Kawada 2014 [33]	CS	Japan	car manufacturing	M	R + N R-N	R + N 99 R-N 686/868	R + N 44.5 R-N 44.3/44.4	SBP DBP	No	5
Kawakami 1998 [34]	CS	Japan	electrical company	M	R + N	^H^ 161/123 ^A^ 280/355 ^P^ 186/178 ^L^ 546/1053	NotR	* SBP * DBP	* age, obesity, exercise, alcohol, education	8
Knutsson 1988 [35]	CS	Sweden	paper and cellulose plants	M	R +N	361/240	43.2/44.8	SBP DBP	No	4
Kubo 2013 [36]	CH (12.7 y)	Japan	industry manufacturing	M	R + N	964/9209	22.3/23.8	SBP DBP * HTN	* age, smoking, alcohol, exercise, BP and BMI at baseline and follow-up	8
Lang 1988 [37]	CS	Senegal	hotel, canning, cotton printing, tobacco, oil, companies	F&M	NS	396/900	M 39.3 ± 9.7 F 35.4 ± 8.8	* SBP * DBP	* age	5
Lercher 1993 [38]	CS	Austria	rural community	F&M	PN	22/147	[25,26,27,28,29,30,31,32,33,34,35,36,37,38,39,40,41,42,43,44,45,46,47,48,49,50,51,52,53,54,55,56,57,58,59,60,61,62]	* SBP * DBP	* age, sex, education, smoking, BMI, other occupational risk factors	8
Lin 2015 [39]	CS	Taiwan	electronics company	F&M	RN	M 447/375 F 118/137	M 31.5/33.8 F 32.5/31.7	SBP DBP	No	4
Marqueze 2013 [40]	CS	Brazil	truck drivers	M	PN	31/26	39.8 ± 6.6	HTN	No	5
Nazri 2008 [41]	CS	Malaysia	semiconductors factory	M	R + N	76/72	31.60/32.32	* HTN	* age, BMI, smoking, exercise, education, marital status, job, working hours and duration	7
Mohebbi 2012 [42]	CS	Iran	long distance drivers	M	PN	3039/3039	[20,21,22,23,24,25,26,27,28,29,30,31,32,33,34,35,36,37,38,39,40,41,42,43,44,45,46,47,48,49,50,51,52,53,54,55,56,57,58,59,60]	SBP DBP	No	4
Morikawa 2007 [43]	CS	Japan	zipper and sash factory	M	R + N	434/712	33.5/36.4	SBP DBP	No+	5
Moy 2010 [44]	CS	Malaysia	medical university	F	R + N	112/268	49.8/49.2	SBP DBP	No	6
Murata 1999 [45]	CS	Japan	copper-smelting plant	M	R + N	158/75	36/36	SBP DBP	No	5
Nagaya 2002 [46]	CS	Japan	manual production, security, transportation	M	R + N	826/2824	45.6/47.1	SBP DBP * HTN	* age, BMI, job, alcohol, smoking, exercise	7
Pimenta 2012 [47]	CS	Brazil	public university	F&M	PN	81/130	[30,31,32,33,34,35,36,37,38,39,40,41,42,43,44,45,46,47,48,49,50,51,52,53,54,55,56,57,58,59,60,61,62]	HTN	No	4
Puttonen 2009 [48]	CS	Finland	population-based	F&M	NS	M 157/555 F 208/623	[24,25,26,27,28,29,30,31,32,33,34,35,36,37,38,39]	SBP DBP	No	5
Sakata 2003 [49]	CH (91-01)	Japan	steel company	M	R + N	2316/3022	NotR	SBP DBP * HTN	* age, BMI, alcohol, smoking, exercise, TC, creatinine, UA GTP, HbA1c	9
Santhanam 2014 [50]	CS	USA	NHANES	F	NS	681/2481	32.9/32.4	SBP HTN	No	4
Sfreddo 2010 [51]	CS	Brazil	nursing staff	F	PN	182/311	36.4/33.1	SBP DBP HTN	No	7
Sookoian 2007 [52]	CS	Argentina	1 factory	F	R + N	474/877	36/34	SBP DBP	No	5
Suessenbacher 2011 [53]	CS	Austria	glass factory	M	R + N	48/47	48/47	HTN	No	5
Tanigawa 2006 [54]	CS	Japan	3 nuclear power plants	M	R + N	253/206	40.4/41.5	SBP DBP	No	6
Virkkunen 2007 [55]	CS	Finland	paper and pulp or oil industries	M	R + N	27/285	[40,41,42,43,44,45,46,47,48,49,50,51,52,53,54,55]	SBP HTN	No	5
Yamasaki 1998 [56]	CS	USA	nursing staff	F	NS	35/58	40.7 [30,31,32,33,34,35,36,37,38,39,40,41,42,43,44,45,46,47,48,49,50,51,52,53,54,55,56,57,58,59]	SBP_AMBP_ DBP_AMBP_	No	6
Ohira 2000 [57]	CS	Japan	nuclear power plant	M	R + N	27/26	30.5/31.8	* SBP_AMBP_ DBP_AMBP_	* age, BMI, alcohol, exercise, anger score	6
Kario 2002 [58]	CS	USA	nursing staff	F	PN	33/54	40/41	SBP_AMBP_ DBP_AMBP_	No	5

CS: cross-sectional study or cross-sectional data; CH: cohort study (dates of baseline and last follow-up or mean years of follow-up); F: female; M: male; SWs: shift workers; DWs: day workers; R + N: rotational shifts including nights; R-N: rotational shifts without nights; PN: permanent night shifts; NS: not specified; NotR: not reported; SBP: systolic blood pressure; DBP: diastolic blood pressure; AMBP: data collected with ambulatory blood pressure monitor; NOS: Newcastle–Ottawa Quality Score; BMI: body mass index; UK: United Kingdom; USA: United States of America; NHANES: National Health and Nutrition Examination Survey; TC: total cholesterol; GTP: gamma glutamyl transferase; HbA1c: glycated hemoglobin; UA: uric acid; ^B^: Factory B; ^C^: Factory C; ^H^: high strain; ^A^: active strain; ^P^: passive strain; ^L^: low strain; * and ** (asterisks): indicate outcomes that were adjusted and the respective confounding variables adjusted; ^i^ when mean age regarding SWs and DWs is not provided, information about the total sample is displayed both as mean ± standard deviation or range (min–max).

## Supplementary Materials

The following are available online at https://www.mdpi.com/article/10.3390/ijerph18136738/s1. Figure S1: Funnel plots and *p*-value (for Egger test) for each outcome; Table S1: Search strategy; Table S2: Key studies excluded at full-text stage, with reasons; Table S3: Newcastle–Ottawa Quality Assessment Score (NOS). Table S4: Results from univariate meta-regression analysis. Table S5. Results from multivariate meta-regression analysis.
