# Supplementary material for: The Impact of Different Types of Shift Work on Blood Pressure and Hypertension: A Systematic Review and Meta-Analysis
Source: Int J Environ Res Public Health. 2021 Jun 23;18(13):6738. doi: 10.3390/ijerph18136738 (PMC8269039; doi:10.3390/ijerph18136738)
Supplement: Supplementary file 1 [file ijerph-18-06738-s001.zip › ijerph-1252540-supplementary.pdf]

International Journal of
Environmental Research and Public Health

MDPI

Systematic Review - Supplementary Materials

The impact of shift work on blood pressure and hypertension: a systematic review and meta-analysis

Sara Gamboa Madeira*, Carina Fernandes, Teresa Paiva, Carlos Santos Moreira and Daniel Caldeira

Supplementary Materials: The following are available online at www.mdpi.com/xxx/s1.

Figure S1: Funnel plots and P-value (for Egger test) for each outcome.

Table S1: Search strategy.

Table S2: Key studies excluded at full-text stage, with reasons.

Table S3: Newcastle-Ottawa Quality Assessment Score (NOS).

Table S4: Results from univariate meta-regression analysis.

Table S5. Results from multivariate meta-regression analysis.

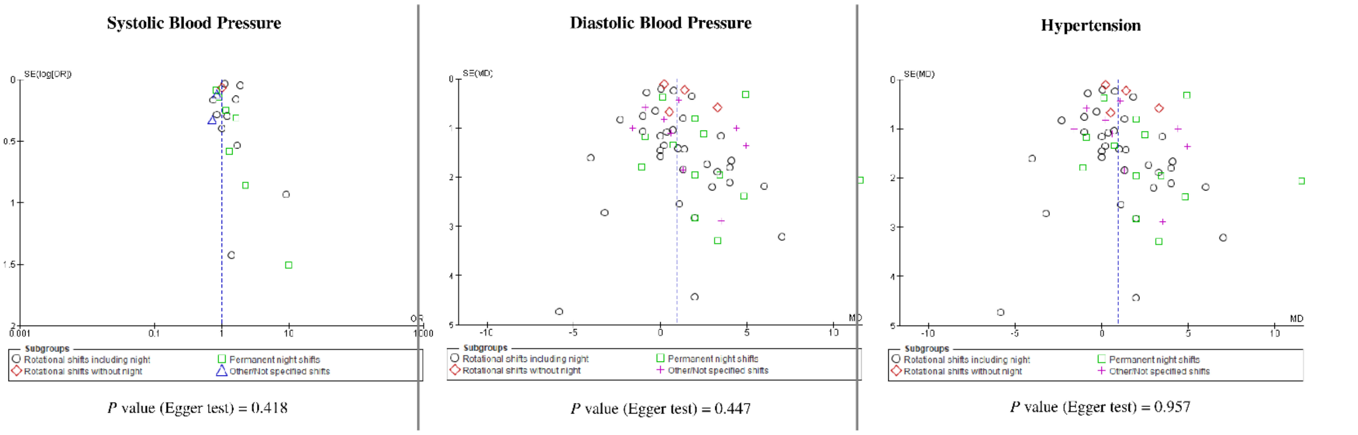

Figure S1. Funnel plots and P-value (for Egger test) for each outcome, systolic blood pressure, diastolic blood pressure, and hypertension.

Table S1. Search strategy

#	MEDLINE (OVID interface)
1	exp Work Schedule Tolerance/
2	exp Shift Work Schedule/
3	exp Sleep Disorders, Circadian Rhythm/
4	(shiftwork* or shift-work* or (shift* adj3 work*)).ab,ti.
5	(nightshift* or nightwork* or night-work or overnight shift* or (night* adj3 shift*) or (night* adj3 work*)).ab,ti.
6	(rotat* adj3 (shift* or work*)).ab,ti.
7	1 or 2 or 3 or 4 or 5 or 6
8	"hypertens*".ab,ti.
9	bloodpressure.ab,ti.
10	((diastolic or systolic or arterial or blood) adj3 pressur*).ab,ti.
11	(BP or SBP or DBP).ab,ti.
12	exp Hypertension/
13	exp Blood Pressure/
14	8 or 9 or 10 or 11 or 12 or 13
15	7 and 14
16	remove duplicates from 15
17	limit 16 to humans

Table S2. Key studies excluded at full-text stage, with reasons.

Author Year	Design	Population	Sex	Reason
Fujino 2006	CH	Multiple	M	Self-reported diagnosis of HTN
Hublin 2010	CH	Multiple	F&M	Self-reported diagnosis of HTN
Lieu 2012	CH	Nurses	F	Self-reported diagnosis of HTN
Ellingsen 2007	CS	Fertilizer plant	M	No definition of HTN diagnosis
Lajoie 2015	CS	Hospital employees	F	BP cut-off of 130/85mmHg
Li 2011	CH	Multiple	F&M	BP cut-off of 130/85mmHg
Pietroiusti 2009	CH	Nurses	F&M	BP cut-off of 130/85mmHg
Tucker 2012	CH	Multiple	F&M	BP cut-off of 130/85mmHg
McNamee 1996	CH	Industrial workers	M	BP cut-off of 160/95mmHg
Peter 1999	CS	Multiple	M	BP cut-off of 160/95mmHg
Sternberg 1995	CS	Bakery	M	No dispersion measure for BP
Ghazanfari 2016	CH	Steel company	M	Apparent population overlap with Fesharaki
Fesharaki 2014	CH	Steel company	M	Apparent population overlap with Fesharaki
Fesharaki 2013	CH	polyacryl corporation	M	Apparent population overlap with Fesharaki
Guo 2015	CS	motor corporation	M	Apparent population overlap with Guo
Kawada 2010	CS	car manufacturing company	M	Apparent population overlap with Kawada
Lin 2009	CS	electronics manufacturing company	F	Apparent population overlap with Lin
Morikawa 1999	CS	zipper and sash factory	M	Apparent population overlap with Morikawa
Murata 2005	CH	copper-smelting plant	M	Apparent population overlap with Murata
Suwazono 2008	CS	steel company	M	Apparent population overlap with Sakata
Tenkane 1997	CS	industrial workers	M	Apparent population overlap with Virkkunen

CS: Cross-sectional study or cross-sectional data; CH: Cohort study; BP: blood pressure; HTN: hypertension; F: Female; M: Male

Table S3. Newcastle-Ottawa Quality Assessment Score (NOS)

NOS Dimension	Selection				Comparability	Outcome			Score Total
Author Year	1)	2)	3)	4)	1)	1)	2)	3)	
Asare-Anane 2015	0	0	0	1	0	2	1	.	4
Attarchi 2012	1	0	1	1	2	2	1	.	8
Balieiro 2014	0	0	0	1	0	2	1	.	4
Biggi* 2008	1	1	1	0	2	1	1	1	8
Bursey 1990	0	0	0	2	0	2	1	.	5
Chan 1993	0	0	0	1	0	2	1	.	4
Chen 2010	0	0	0	1	0	2	1	.	4
De Bacquer 2009	1	0	1	1	0	2	1	.	6
DeGaude-maris 2011	1	0	1	1	0	2	1	.	6
Di Lorenzo 2003	1	0	1	1	0	2	1	.	6
Ely 1986	1	0	1	1	0	2	1	.	6
Ohlander 2015	1	0	0	2	2	2	1	.	8
Fesharaki 2014	1	0	0	2	2	2	1	.	8
Guo 2013	1	0	1	1	0	2	1	.	6
Ghiasvand 2006	0	0	0	1	2	2	1	.	6
Ishizuka 1993	0	0	0	2	0	2	1	.	5
Jermendy 2012	0	0	0	1	0	2	1	.	4
Kantermann 2013	0	0	0	1	0	2	1	.	4
Kawabe 2014	1	0	0	1	0	2	1	.	5
Kawada 2014	1	0	0	1	0	2	1	.	5
Kawakami 1998	1	0	1	1	2	2	1	.	8
Knutsson 1988	0	0	0	1	0	2	1	.	4

Kubo* 2013	1	1	1	1	2	1	1	0	8
Lang 1988	1	0	0	1	0	2	1	.	5
Lercher 1993	1	0	1	1	2	2	1	.	8
Lin 2015	0	0	0	1	0	2	1	.	4
Marqueze 2013	1	0	0	1	0	2	1	.	5
Nazri 2008	1	0	0	1	2	2	1	.	7
Mohebbi 2012	0	0	0	1	0	2	1	.	4
Morikawa 2007	1	0	0	1	0	2	1	.	5
Moy 2010	1	0	1	1	0	2	1	.	6
Murata 1999	1	0	0	1	0	2	1	.	5
Nagaya 2002	1	0	0	1	2	2	1	.	7
Pimenta 2012	0	0	0	1	0	2	1	.	4
Puttonen 2009	1	0	0	1	0	2	1	.	5
Sakata* 2003	1	1	1	1	2	1	1	1	9
Santhanam 2014	0	0	0	1	0	2	1	.	4
Sfreddo 2010	1	1	1	1	0	2	1	.	7
Sookoian 2007	1	0	0	1	0	2	1	.	5
Suessen-bacher 2011	0	0	0	2	0	2	1	.	5
Tanigawa 2006	1	0	0	2	0	2	1	.	6
Virkkunen 2007	1	0	0	1	0	2	1	.	5
Yamasaki 1998	1	0	1	1	0	2	1		6
Ohira 2000	0	0	0	1	2	2	1	.	6
Kario 2002	1	0	0	1	0	2	1	.	5

Cohort studies: selection: 1) Representativeness of the exposed cohort; 2) Selection of the non exposed cohort; 3) Ascertainment of exposure; 4) Demonstration that outcome of interest was not present at start of study; comparability: 1) Comparability of cohorts on the basis of the design or analysis outcome: 1) Assessment of the outcome; 2) Was follow-up long enough for outcomes

to occur; 3) Adequacy of follow-up; Cross-sectional studies [9]:selection: 1)Representativeness of the sample; 2) Sample size; 3) Non-respondents; 4) Ascertainment of the exposure; comparability: 1) Confounding factors are controlled; outcome: 1) Assessment of the outcome; 2) Statistical tests

Table S4. Results from univariate meta-regression analysis.

Outcome<br>Covariates	Coeff.	Sdt. Err.	t	P>|t|	95%CI lower	95%CI superior	N
**Systolic BP**							
Male	-1.369818	0.7570138	-1.81	0.076	-2.886907	0.147272	57
Constant	2.209518	.6203227	3.56	0.001	.9663635	3.452673	
Age	-.0055317	.0458452	0.12	0.904	-0.097615	0.086551	52
Constant	1.685725	1.820996	0.93	0.359	-1.971853	5.343303	
Smoking	-2.827923	2.216863	-1.28	0.209	-7.308369	1.652524	42
Constant	2.405204	.9814952	2.45	0.019	.421528	4.388879	
BMI	-.2229164	.2352946	-0.95	0.349	-0.699245	0.253413	40
Constant	7.172097	5.857529	1.22	0.228	-4.685851	19.03004	
**Diastolic BP**							
Male	-.8186015	.5205969	-1.57	0.122	-1.863256	0.226053	54
Constant	1.152487	.4305318	2.68	0.010	.2885619	2.016412	
Age	-.0016895	.0305026	-0.06	0.956	-0.063019	0.059640	50
Constant	.7217071	1.222945	0.59	0.558	-1.737188	3.180603	
Smoking	-1.696456	1.489377	-1.14	0.262	-4.709004	1.316093	41
Constant	1.28408	.6549514	1.96	0.057	-.0406845	2.608844	
BMI	.0177833	.1553774	0.11	0.910	-0.2973366	0.332903	38
Constant	.2409512	3.874287	0.06	0.951	-7.616467	8.09837	

Outcome/target variables included SBP and DPB; SBP: systolic blood pressure; DBP: diastolic blood pressure; BMI: body mass index; Coeff: regression coefficient; Sdt. Err.: Standard Error; t:t value; P>|t|: 2-tailed p-value; 95%CI: 95% confidence intervals (lower and superior limits); N: number of estimates included in each analysis.

Table S5. Results from multivariate meta-regression analysis.

Outcome<br>Covariates	Coeff.	Sdt. Err.	t	P>∣t∣	95%CI lower	95%CI superior	N
**Systolic BP**							
Male	1.07127	1.63436	-0.66	0.520	-4.480485	2.337945	
Age	-.0032181	.0684423	-0.05	0.963	-0.145986	0.139551	
Smoking	-1.715447	3.822679	-0.45	0.658	-9.689417	6.258522	
BMI	-.1138207	.3470926	-0.33	0.746	-0.837843	0.610202	
Constant	6.461052	8.352878	0.77	0.448	-10.96275	23.88485	25
**Diastolic BP**							
Male	-1.769282	1.105564	-1.60	0.125	-4.075447	0.536883	
Age	-.0074459	.0446607	-0.17	0.869	-0.100607	0.085715	
Smoking	-.3756096	2.47611	-0.15	0.881	-5.540685	4.789466	
BMI	.1840371	.2231893	0.82	0.419	-0.281528	0.649602	
Constant	-1.89018	5.388936	-0.35	0.729	-13.13130	9.350943	25

Outcome/target variables included SBP and DPB; SBP: systolic blood pressure; DBP: diastolic blood pressure; BMI: body mass index; Coeff: regression coefficient; Sdt. Err.: Standard Error; t:t value; P>∣t∣: 2-tailed p-value; 95%CI: 95% confidence intervals (lower and superior limits); N: number of estimates included in each analysis.
